# A cluster randomised controlled trial evaluating the effectiveness of a structured pulmonary rehabilitation education programme for improving the health status of people with chronic obstructive pulmonary disease (COPD): The PRINCE Study protocol

**DOI:** 10.1186/1471-2466-11-4

**Published:** 2011-01-18

**Authors:** Kathy Murphy, Dympna Casey, Declan Devane, Adeline Cooney, Bernard McCarthy, Lorraine Mee, Martina Nichulain, Andrew W Murphy, John Newell, Eamon O' Shea

**Affiliations:** 1 School of Nursing and Midwifery, National University of Ireland, Galway, Ireland; 2 Clinical Research Facility, National University of Ireland, Galway, Ireland; 3 Department of General Practice, National University of Ireland, Galway, Ireland; 4 HRB Clinical Research Facility and School of Mathematics, Statistics and Applied Mathematics, National University of Ireland, Galway, Ireland; 5 Head of the Economics Department, National University of Ireland, Galway, Ireland

## Abstract

**Background:**

A key strategy in improving care for people with chronic obstructive pulmonary disease (COPD) is the provision of pulmonary rehabilitation programmes. Pulmonary rehabilitation programmes have been successful in improving patients' sense of dyspnoea and Health Related Quality of Life. However, the effectiveness of structured education pulmonary rehabilitation programmes delivered at the level of the general practice on the health status of people with COPD remains uncertain and there is a need for a robust and fair assessment of this. The PRINCE study will evaluate the effectiveness of a Structured Education Pulmonary Rehabilitation Programme (SEPRP), delivered at the level of the general practice, on the health status of people with COPD.

**Methods/Design:**

The PRINCE Trial is a two-armed, single blind cluster randomised trial conducted in the primary care setting in Ireland. Randomisation to control and intervention is at the level of the General Practice. Participants in the intervention arm will receive a SEPRP and those allocated to the control arm will receive usual care. Delivery of the SEPRP will be by a practice nurse and physiotherapist in the General Practice (GP) site. The primary outcome measure of the study will be health status as measured by the Chronic Respiratory Questionnaire (CRQ). Blinded outcome assessment will be undertaken at baseline and at twelve-fourteen weeks after completion of the programme. A comparison of outcomes between the intervention and control sites will be made to examine if differences exist and, if so, to what extent between control and experimental groups. Sample size calculations estimate that 32 practices with a minimum of 10 participants per practice are required, in total, to be randomised to control and intervention arms for power of at least 80% with alpha levels of 0.05, to determine a clinically significant change of 0.5 units in the CRQ. A cost effectiveness analysis will also be conducted.

**Discussion:**

The results of this trial are directly applicable to primary care settings in Ireland. Should a SEPRP delivered by practice nurses and physiotherapists in primary care be found to be effective in improving patients' sense of dyspnoea and HRQoL, then the findings would be applicable to many thousands of individuals in Ireland and beyond.

**Trial registration:**

ISRCTN: ISRCTN52403063

## Background

By 2030, COPD will rank seventh in the worldwide burden of disease [1] and will be the third most frequent cause of death [2]. Few countries, however, have good population-based data on COPD and estimates of the prevalence of COPD differ widely, depending on the criteria that are used. Halbert et al. [3] report prevalence rates of 0.23% to 18.3% in Europe and North America with typical rates of 4 to 10%. The Burden of Obstructive Lung Disease (BOLD) initiative found that the prevalence rates of stage II or higher COPD was 10.1% greater than that reported in previous studies suggesting that prevalence rates of COPD may be underestimated [4]. There is little accurate data available on the prevalence of COPD in Ireland. A useful comparison may, however, be made with data from the United Kingdom where it is estimated that the rate of COPD in the population attending GPs ranges from 2% - 4%.

COPD is also the leading cause of workdays lost in the European Union [5]. In Ireland, the Economic and Social Research Institute [6] found that 20% of inpatient hospital bed days were due to COPD and in those aged over 65 years, COPD accounted for nearly a third of respiratory inpatient cases. This implies that if illness progression can be delayed and acute exacerbations of COPD prevented or reduced then significant alleviation of the economic and clinical burden could be achieved [7,8].

One of the key strategies in improving care for people with COPD is the provision of pulmonary rehabilitation (PR) programmes. PR programmes utilise a multidisciplinary approach and typically consist of a patient assessment, exercise training, education and psychosocial support [9]. They have been successful in improving patients' sense of dyspnoea and Health Related Quality of Life (HRQoL) [10-12]. PR programmes emphasise self-care and self-management. However, the extent to which these programmes meet the criteria for a structured education programmes [13] is unclear. Structured education programmes typically consist of a clear philosophy and a written, structured curriculum and include transparent quality assurance mechanisms. They are delivered by educators who have been trained to deliver the programme. Such programmes are more effective in promoting self-management in chronic conditions than unstructured education programmes [13].

PR programmes have been predominantly hospital or home based, although provision in the primary health care setting has been recommended [14,15]. To date, only one trial evaluating the effects of pulmonary rehabilitation on exercise tolerance and quality of life in people with COPD in the primary care setting has been identified [16]. This study included participants with asthma (n = 23) and COPD (n = 43). The pulmonary rehabilitation programme was delivered by a physiotherapist and included exercise classes, breathing techniques and relaxation activities. Of the 99 participants randomised, 23 subsequently withdrew and their outcomes were unavailable for analysis. Of those remaining, outcome data on quality of life (QoL) as measured by the dyspnoea, fatigue, emotion and mastery components of the Chronic Respiratory Disease Questionnaire (CRDQ) are available for only 23 participants. This study concluded that pulmonary rehabilitation for patients with COPD or asthma delivered in local practices improves exercise tolerance and quality of life.

The effectiveness of structured education pulmonary rehabilitation programmes delivered at the level of the general practice on the health status of people with COPD remains uncertain and there is a need for a robust and fair assessment of this. The PRINCE study will provide this assessment and will also give people with COPD an opportunity to voice their experiences of living with COPD and their approach to self-management.

Our proposed intervention, a structured education pulmonary rehabilitation programme, is delivered at the level of the general practice and is provided to a group of people with COPD rather than to individual patient participants. Therefore, the proposed trial is clustered at the GP practice level.

## Aim

The primary aim of the PRINCE study is to evaluate the effectiveness of a structured education pulmonary rehabilitation programme (SEPRP), delivered at the level of the general practice, on the health status of people with COPD. The trial has four main objectives:

1. To develop a comprehensive SEPRP specifically orientated toward delivery by GP practice nurses to people with COPD in their local communities.

2. To evaluate the effectiveness of the SEPRP within the context of a cluster randomised trial.

3. To understand participants' perceptions and experiences of 'COPD', its impact on their lives and their approach to self-management.

4. To evaluate the cost effectiveness of the provision of a SEPRP for people with COPD.

## Methods

## Design

The PRINCE Trial is a two-group, single blind cluster randomised trial conducted in the primary care setting in Ireland. Randomisation to control and intervention is at the level of the General Practice. Effective randomisation of a sufficiently large sample of participants to control and experimental groups ensures that there are no systematic differences between control and intervention groups in known (e.g. motivation) and unknown factors that may affect the outcome [17]. Participants allocated to the intervention group will receive a SEPRP and those allocated to the control group will receive usual care. Delivery of the PR programme will be by a practice nurse and physiotherapist in GP sites. Blinded outcome assessment will be undertaken at baseline and at twelve-fourteen weeks after completion of the programme. A comparison of outcomes between intervention and control sites will be made to examine if differences exist and, if so, to what extent between control and experimental groups. Randomisation to the PRINCE study began in September, 2009 and outcome assessment is due to end in February 2011.

## Eligibility Criteria

### Participants: GP practices (Clusters)

GP practices across the North Western, Western and Midland Health Service Executive (Ireland) regions who meet the following eligibility criteria are eligible to participate:

1. Supported by a practice nurse;

2. Practice supported by a computerised patient (medication recording) system;

3. Commitment on the part of the practice team to participate in the proposed study;

4. Have a patient population of more than 2500;

5. Participation by a minimum of 10 consenting patients meeting the eligibility criteria.

### Participants: Patients

Primary health care in Ireland is relatively under resourced and is characterised by poor infrastructure and limited interdisciplinary teamwork [18]. General Practitioners tend to operate independently to other primary health care services leading to a fragmented service delivery. Hence, most primary health care settings in Ireland have not implemented a strategy to proactively diagnose COPD as a part of routine practice. For this reason, potential patient participants will have either an existing diagnosis or be suspected of having COPD. COPD will be suspected when patients are receiving drug treatment for respiratory conditions, have a history of tobacco use, a chronic cough, regular sputum production, frequent winter bronchitis, breathlessness or any combination of these. Presence of COPD will be confirmed at baseline assessment by spirometry.

Diagnosis is confirmed with a post Bronchial Dilator FEV1/FVC ratio of less than 70%* unless BMI > 30 in which case FEV1/FVC ratio > 70% are acceptable provided other criteria are fully met and with a post bronchial dilator predicted value of FEV1 ≥ 30% and ≤ 80% (after Global Initiative for Chronic Obstructive Lung Disease (GOLD) 2008 [14]). The decision to adjust the spirometry inclusion criteria for individuals with a BMI > 30 is based on research undertaken by Wannamethee et al [19] who demonstrated that BMI was positively associated with FEV1:FVC.

Patients must be able to converse in and read English as initial delivery of the SEPRP will only be available in English. Patients must also understand the study and give informed consent. Patients will not be excluded if they have previously attended pulmonary rehabilitation programmes and all patients will remain under the care of their GP and/or respiratory physician.

Patients will be excluded from the study if they possess any significant underlying co-morbidities or mental health problems (based on the recorded judgement of practice staff), which are likely to impair their capacity to successfully participate in or assimilate new information as part of the rehabilitation programme or which may pose a risk to their health. Further detail on patient eligibility assessment is provided in Table 1.

**Table 1:** Patient exclusion criteria

**History of**	**Currently:**
• Unstable cardiovascular disease	
ο unstable angina	Suspected or on treatment of relevant and/or current infectious disease (e.g. TB)
ο unstable aortic valve disease	• In the 3^rd ^Semester of Pregnancy
ο unstable pulmonary hypertension	• Haemoptysis of unknown origin
• Pneumothorax	
• Thoracic or Cerebral Aneurysms	**Having had within:**
• Musculoskeletal or neurological disorders that prevent gentle exercise	1 Month:
• Severe cognitive impairment	• Myocardial infarction^3^
• Severe psychotic disturbance	• Eye Surgery
• Detached Retina	3 Months
• Relative contraindications include	• Abdominal or thoracic surgery
ο a'resting heart rate of more than 120^3^	
ο a'systolic blood pressure of more than 180 mm Hg	
ο a'diastolic blood pressure of more than 100 mm Hg	

## Interventions

### Control Group (usual care)

The control group will receive 'usual care'. While the research team acknowledge the complexity and potential heterogeneity of 'usual care', substantial effort will be made to describe clearly the components of 'usual care' for COPD patients through information gathered from structured interviews with GPs, Practice Nurses, documentary analysis of participants health records and through interviews with participants. Practice nurses in the control group will be offered the SEPRP following the completion of post-programme data collection.

### Experimental Intervention

The experimental group will receive the SEPRP, which focuses on improving the self-management skills of persons with COPD. Structured education programmes are defined as planned programmes that are: "...comprehensive in scope, flexible in content, responsive to an individual's clinical and psychological needs and adaptable to his or her educational and cultural background" [13] (pg 27).

Across the literature it is clear that structured education should aim to: empower, inform and support self-management skills. The Department of Health [20] suggests that the aim of education is to improve knowledge and skills, enabling the person to take control of their own condition and to integrate self-management into their daily lives. They suggest that a supported self-care service model is most appropriate to chronic disease management. NICE [13] identify five key criteria for high quality structured education programmes (i) programmes should have a structured written curriculum (ii) a patient centred philosophy (iii) be delivered by trained educators (iv) be quality assured and (v) audited. The SEPRP was developed in accordance with these key criteria and in consultation with people with COPD, experts in pulmonary rehabilitation, general practitioners and practice nurses. The SEPRP was also informed by a detailed analysis of the literature in relation to pulmonary rehabilitation programmes.

The programme has been reviewed and validated by educational experts who were asked to focus on adult learning and empowerment and experts in pulmonary rehabilitation and COPD who were asked to focus on the appropriateness and comprehensiveness of content. The SEPRP has a course philosophy, a detailed curriculum, course materials and a training programme for all educators. It is founded on the principles of empowerment, self-efficacy and behavioural change theory and its development was guided by the Stages of Change/Transtheoretical Model of Behaviour Change [21]. Central to the programme is empowering people with COPD to manage their care well; consequently the programme delivery adopts a patient-centred approach with a focus on priority setting and problem solving. It consists of an eight-week programme with a two-hour session each week (16 hours total duration) delivered jointly by a practice nurse and physiotherapist. An overview of programme content is presented in Table 2.

**Table 2:** Content of SEPRP

Week	Content	Educator
**Week 1**	Setting the scene	Practice Nurse
	Introduction to exercise	Physiotherapist

**Week 2**	Managing medications	Practice Nurse
	Exercise programme	Physiotherapist

**Week 3**	Exercise programme	Physiotherapist
	Exercise programme	Physiotherapist

**Week 4**	Managing breathlessness	Practice Nurse
	Exercise programme	Physiotherapist

**Week 5**	Knowing and managing your symptoms	Practice Nurse
	Exercise programme	Physiotherapist

**Week 6**	Recognising and managing acute exacerbations	Practice Nurse
	Exercise programme	Physiotherapist

**Week 7**	Exercise programme	Physiotherapist
	Managing stress and anxiety	Practice Nurse

**Week 8**	Exercise programme	Physiotherapist
	Where to now?	Practice Nurse

Support following the intervention is thought to be highly significant in adherence to the programme principles [22]. For this reason, practice nurses will provide ongoing advice and support to participants as required throughout the intervention. In addition, participants in the intervention will also be followed-up formally at 4 weeks and at 12-14 weeks after completion of the SEPRP.

The 4-week follow up is by telephone and aims to provide positive reinforcement to those adhering to the programme, identify any barriers preventing participants from adhering to the programme and discuss strategies to overcome these. At the 12-14 weeks follow up, participants will be asked to attend a 1-hour group session. This session will be delivered by the practice nurse. It builds on the eight-week structured education programme and aims to further empower inform and support persons with COPD to continue self-managing their COPD. Similar to the eight-week structured education programme, this follow up programme is underpinned by the principles of adult learning, an empowerment philosophy and the transtheoretical model of change. The focus of this programme is to facilitate clients to self evaluate their progress, appraise their achievements, problem solve and plan for the future.

It should be noted that we are not seeking to evaluate individual components of SEPRP but rather the entire programme. If there is a beneficial effect in outcomes for participants in the intervention arm we will be able to conclude that SEPRP is effective but will not be able to conclude what specific components of SEPRP contributed most to such effectiveness.

#### Practice Nurse Preparation Programme

Practice Nurses will attend a three day Practice Nurse Preparation Programme (PNPP) to prepare them to deliver the SEPRP.

The programme has four main components: (1) understanding the philosophy of the SEPRP, (2) principles of adult learning and effective management of small groups, (3) an introduction to programme materials and approaches to its implementation and (4) assessment of competence in delivery. Sessions are interactive and geared towards meeting participants' learning goals and needs. This programme was reviewed and validated by outside experts and was also piloted. The PNPP will be delivered in two stages. The initial two-days will focus on preparing practice nurses to deliver the SEPRP and on building confidence (Components 1, 2, 3). On the third day, held a week later, participants will role play delivering to their peers an assigned element of the structured education programme (Component 4). The research team will observe these presentations and feedbacks to each participant on their performance including their adherence to lesson plans and activities within the SEPRP curriculum. Providing participants with an opportunity to present a segment of the SEPRP will increase their confidence and skills for delivering the SEPRP. To ensure standardisation of programme content and delivery, members of the research team will deliver all PNPP training programmes. Practice nurses will be audited when delivering the SEPRP programmes in weeks 4 or 5 to ensure adherence to programme principles and content. An action plan will be developed and followed up as required.

#### Physiotherapist Preparation Programme

Physiotherapists will attend a one-day Physiotherapist Preparation Programme (PPP) focused on preparing physiotherapists to deliver the structured education programme. Programme content aims to prepare participants to: (1) understand the philosophy of the SEPRP, (2) understand and apply the principles of adult learning and effective management of small groups, (3) understand programme materials and approaches to its implementation and (4) deliver the exercise component of the programme as planned. Sessions will be interactive and geared towards meeting participants' learning goals and needs. There are 10 exercise stations each of 3 minutes duration focused on building exercise capacity, and tailored to each participant's individual needs. These exercises will also be repeated at home and recorded by participants in a home exercise diary.

Again to ensure standardisation of programme content and delivery, members of the research team will deliver all PPP programmes. Physiotherapists will also be audited in their facilitation of the SEPRP programmes in weeks 4 or 5 to ensure adherence to programme principles and content. Similar to the PNPP, an action plan will be developed and followed up if required.

All educators (practice nurses and physiotherapists) will be supported in the delivery of the programme over the eight weeks by having recourse to members of the research team for purposes of clarification on training materials and in identifying sources of information in response to specific patient queries.

## Outcome measures

Outcome measures will be collected from participants in both intervention and control groups at baseline (following consent, prior to randomisation and group allocation) and at 12-14 weeks post completion of the 8-week programme or at an equivalent time for the control group. We acknowledge that a longer follow-up period would be desirable but funding does not permit follow-up beyond this.

Each participating practice will be assigned a Research Assistant responsible for outcome assessment on all participating patients within that practice. To enhance the quality of measurements, all Research Assistants will undergo a one-day Research Assistant Preparation Programme consisting of:

i) Training on the procedures and protocols surrounding the delivery, assessment and recording of all outcome measurements;

ii) Discussion on adverse events and their management;

iii) Simulated completion of all data collection instruments and forms;

iv) Basic Life Support training (for those requiring certification);

### Primary

The primary outcome measure of the study will be health status as measured by the Chronic Respiratory Questionnaire (CRQ) [23]. This instrument was developed following interviews with people who had COPD [23]. The questionnaire has four domains: dyspnoea, fatigue, emotional function and mastery.

The validity and reliability of this tool was first investigated by Wijkstra et al. [24] who concluded that the CRQ was a valid and reliable tool. This conclusion was also confirmed by Curtis and Patrick [25] who found that the CRQ was reliable, valid and responsive to change. In addition, they claim that the CRQ is more sensitive to change than other generic health status instruments.

### Secondary

1. Incremental Shuttle Walking Test;

2. Self-Efficacy for Managing Chronic Disease 6-Item Scale [26];

3. Economic analysis specific:

a) EuroQol EQ-5D [27];

b) Utilisation of health care Service Utilisation of health care Service. For variables, i to v, we are dependent on practice notes for their determination. Based on our previous experience [28,29] we consider these to be reliable and valid:

i) Hospital admissions/length of stay;

ii) Attendance at the emergency department;

iii) Outpatient attendances;

iv) Attendance at/by GP;

v) Attendance at/by Practice Nurse;

vi) Attendance at/by Public Health Nurse;

vii) Attendance at/by Physiotherapy;

viii) Attendance at/by Social Worker;

ix) Attendance at/by Dietician;

x) Outpatient attendances;

xi) Attendance at/by consultant;

xii) Utilisation of Home help;

### Demographic variables

Data will be collected from each patient participant on current medication use, smoking history, education level, marital and employment status, level of social engagement and level of support with activities of daily living.

## Sample Size Estimation

We used methods for standard sample size estimates for trials that randomised at the level of the individual [30] adjusting for clustering by inflating sample size estimates by the design effect given by $1+(\bar{n}-1)\rho$, where $\bar{n}$ is the average cluster size, and ρ is the estimated intraclass correlation coefficient (ICC) [31]. Sample size estimates are based on the primary outcome of health status as measured by the CRQ [23], expressed as the mean rate difference between intervention and control groups.

Previous work suggests 0.5 as the minimal clinically important mean difference for all four dimensions of the CRQ [32] and estimates of standard deviations of 1.145 (given as arithmetic mean of 0.88, 1.06, 1.24 and 1.40 for the Dyspnoea, Fatigue, Emotion and Mastery dimensions of the CRQ respectively) [33]. Using these values and the upper value of 0.05 from a range of ICC values identified within studies involving the older person in primary care, [34] calculations indicate that 32 practices with a minimum of 10 participants per practice are required, in total, to be randomised to control and intervention arms for power of at least 80% with alpha levels of 0.05. This allows for participant loss to follow-up of 20% plus a loss of 4 practices. ICC values lower than 0.05 would increase the power of the study.

## Randomisation

### Random allocation sequence generation

Randomisation is at the level of the general practice (cluster). The random allocation sequence will be generated using a computer generated random number list [35]. To minimise the time delay between practices agreeing to participate and the commencement of the training programme for practice nurses and the implementation of the intervention for experimental clusters and patients therein, practices will be randomised in blocks of clusters when all patient participants in the block of clusters have been recruited. We propose to randomise clusters in four groups of clusters on a 1:1 ratio. This approach is recognised as a legitimate means of overcoming long delays between recruitment and implementation of the intervention in cluster trials [36].

### Allocation concealment

Concealment of group allocation will be achieved by giving the responsibility for allocation sequence generation and group allocation to a researcher independent of the study and its investigators.

### Implementation

The research team will provide the independent researcher with a list of the first group of participating practices that meet the eligibility criteria and consent to participate. The independent researcher will then (i) consecutively number all participating practices in this group (ii) generate the random allocation sequence as detailed (iii) assign practice to group allocation and (iv) notify the PRINCE Project Manager of the practice allocations. The same allocation procedure will be followed for subsequent groups.

### Blinding

Patient participants within each participating practice will be recruited prior to random allocation of practices to control and intervention groups. Because of the nature of the intervention, it is not possible to blind participants (GP Practices or clients) to practice group allocation. Outcome assessment will be blinded to group allocation by (i) blinding Research Assistants responsible for outcome assessment to the group allocation of participating practices and patients with any exceptions recorded and (ii) having data analysis performed by researchers and statisticians blinded to group allocation by providing database of outcomes with groups identified numerically only.

## Quantitative Analysis

The analysis will be based on the GP Practice with the patient as the unit of analysis whilst accounting for the intracluster correlation coefficient. Analyses will be by intention to treat. Quantitative data will be analysed, in aggregate, using The Statistical Package for the Social Sciences (SPSS, v17) and R ([37], 2.11). Data will be entered into SPSS, coded, cleaned and locked before any analyses are made. Only members of the research team who needed access to the database to fulfil their roles within the study were granted access to the database. Funders did not have access to the database at any time. Demographic characteristics of the practices and the participants within the practices will be described using percentages, measure of central tendency (means or medians) and measures of variation (standard deviations or ranges). Comparability of randomised groups by socioeconomic and other categorical baseline variables will be by the chi-square (*X*^2^) test.

Whilst every effort will be made to exclude all confounders at the design phase of the study, this is not always possible. The analysis of data will therefore include the search for and control of nuisance variables for which adjustments had not been made. In particular, a mixed effects model will be used to model the longitudinal change in the mean scores for each of the four dimensions of the CRQ primary response while adjusting for explanatory variables and baseline CRQ as needed and incorporating random effects to model the homogeneity within cluster and the correlation within patient across time. The Akaike information criterion (AIC), for linear mixed-effects models in the analysis of clustered data, will be used to decide between candidate models [38]. The significance level will be set at α = 0.05 for all analyses.

## Rigour

### Treatment fidelity

Threats to treatment fidelity will be minimised by providing the SEPRP, PNPP and PPP programmes within the context of comprehensive, structured curriculum documents and through delivery of training by members of the research team. Each programme will be audited in week 4 or 5 to assess adherence to programme principles. An action plan will be implemented if there are any concerns regarding adherence to programme content and principles. Providing PNPP participants with structured feedback (Component 4 above) will also enhance treatment fidelity.

### Data Integrity

Data integrity will be enhanced by having data collection performed by a small number of trained research assistants and by adherence to assessment protocols.

Errors will be logged and remedial strategies implemented as required. The central study processes (e.g. eligibility assessment, outcome assessment etc.) will be kept under review to add to the rigour of the study.

Single data entry into SPSS will be performed with visual verification of a sample of records from the data set created from the single entry using a continuous sampling plan (CSP-1) (after [39]). A CSP-1 gives the number of successive records with no data entry errors that must be inspected *i *before a random sample fraction *f *of records will begin. Whenever an error is found, the error is corrected and the successive record checking using i is repeated.

### Adverse events

All adverse events that occur after informed consent will be recorded on an Adverse Event Recording Form (AERF) and reported to the patients General Practitioner within the respective practice and to the principal investigators. The PRINCE team have provisionally defined an adverse event as 'Any acute alteration in the patient's physiological condition'. A protocol for dealing with adverse episodes has been developed and is included as part of the training programme for the research assistance and practice nurses.

All research assistants will be trained in basic life support and will carry emergency resuscitation equipment to all patient assessments. Physiotherapists will also bring resuscitation equipment to each programme session.

## Pilot study

A pilot study will be conducted with one GP practice. This pilot will be used to identify problems with the research design/processes; refine data collection and analysis; assess adequacy of data sources; examine selection and enrolment processes; and assess the participants' perspective on participating. We will not include data from the pilot period in the main analyses of the trial.

## Embedded Qualitative Study

## Introduction and design

To enhance the overall design of the study a qualitative strand was added. This approach is described as an "embedded design" [40]. Embedded designs are used when the inclusion of qualitative data would be helpful in addressing secondary research questions within the larger quantitative study [40]. The overall aim of the embedded qualitative study is twofold: (1) to examine the *proces*s of the intervention and (2) to define *usual care*. The specific aims of this element of the study are to:

• understand participants perceptions of COPD and its impact on their day-to-day lives;

• determine the components of usual care for participants allocated to the control group;

• understand individuals approach to and experiences of managing COPD prior to the SEPRP;

• understand the impact that the SEPRP may have on participants' self-management skills;

• gain insights into the factors that govern adherence to programme principles.

## Grounded Theory

A grounded theory methodology (after Corbin and Strauss [41]), was chosen to guide the qualitative element of this study because of its focus on how people respond to and act towards the problems they encounter [41,42]. Grounded theory aims to produce a theory to "fit" (i.e. has relevance) the situation, aid understanding and guide action and practice [41]. These are important considerations when studying health related problems. Grounded theory 'discovers' theory through systematic data collection and analysis [41].

## Data collection Methods

In-depth, one-to-one interviews (guided by an interview guide) will be the primary method of data collection. Participants will be interviewed on three occasions (1) prior to the participating in the SEPRP programme (2) within 2-6 weeks of completing the SEPRP and (3) three months after completion of the programme. This longitudinal design will enable understanding of the reasons for participant's changes (or not maintaining change) in self-management skills and their exercise regimes over time.

In addition, observational data (guided by an observation schedule) will be collected from one session of the SEPRP for each group from which the sample is drawn. This data will help set the interviews in context. In addition to providing contextual data, the literature suggests that the group experience (the synergistic effect of the group) may be an important factor in behaviour maintenance. Observational sessions will be of two hours duration i.e. observing a one-hour SEPRP delivered by the practice nurse (detailed in Table 2) and one-hour delivered by the physiotherapist where participants complete an exercise circuit.

## Sampling in the intervention group

Twenty participants will be recruited from those allocated to the intervention group using purposive sampling initially and theoretical sampling later on (guided by data analysis) to ensure diversity of experiences. Theoretical sampling is the process of simultaneously collecting, coding and analysing data to generate theory [41]. Grounded theorists do not seek representativeness of people or events but the representativeness of "concepts" [41]. Sampling decisions are driven by "... *concepts that emerged from analysis and that appear to have relevance to the evolving theory" *[43]. To ensure participant diversity in the initial purposive sample, the following inclusion criteria will be used:

• *Severity of COPD *- people with moderate to severe COPD (as defined by the study protocol) will be included;

• *GP practice size *- people attending GP Practices with (1) between 2,500 and 5,000 patients and (2) over 5,000 patients will be included;

• *Gender *- both men and women will be included;

• *Location of practice *- urban and rural practice settings will be included.

Purposive sampling will be superseded by theoretical sampling as the study progresses. Theoretical sampling may indicate the need to include more diverse participants and will evolve as the study progresses.

## Sampling in the control group

Structured qualitative interviews will be conducted with ten patient participants and ten practice staff (either practice nurses or GPs) from the control group. These participants will be interviewed on one occasion only. This data will be used to define and describe usual care.

## Sampling of staff delivering the SERPS

All practice nurses and physiotherapists who delivered the SEPRP will be interviewed. This interview will focus on their experience of delivering the SEPRP. This data will provide contextual information and insight into *how *staff delivered and their *experience *of delivering the SERPS.

## Analysis

The constant comparative technique [41] will be used to analyse data. Data from each group will be analysed individually (within group) and, in the case of participants who completed the SEPRPS i.e. completed three interviews (across group), to explore the impact of time on the impact of the SEPRPS. Data will be analysed in stages (e.g. all the interviews from a practice) reflective of recruitment phases. This approach enables data analysis to guide data collection and sampling decisions (theoretical sampling).

## Economic Evaluation

The economic evaluation explores the cost effectiveness of the PRINCE intervention relative to usual care for patients with COPD in Ireland. This involves the identification, measurement and valuation of all relevant costs and benefits. The time horizon is equivalent to that of the follow up for the cluster randomised controlled trial. Retrospective data on resource use is collected at baseline and 22 weeks later at the end of the study. Although the time period is relatively short, the hypothesis is that there will be positive changes in resource use for the intervention group, most likely a reduction in the probability of being hospitalised, which will yield significant levels of savings [44]. Incremental cost effectiveness analysis is used, whereby we estimate the differences in mean cost and mean effectiveness across treatment arms, and relate the difference in mean cost to the difference in mean effectiveness. Uncertainty surrounding the incremental results is assessed using cost effectiveness acceptability curves, which will present the weight of evidence in favour of intervention relative to control.

With respect to costs, we consider both the healthcare resource requirements and the private patient expenses associated with each treatment group.

Specifically, we collect data on three areas of resource use alongside the trial: Intervention costs, which include all the resources required to organise and implement the Structured Pulmonary Rehabilitation Programme in clinical practice, Other healthcare services costs, which includes the use of all primary and secondary care over the course of the trial, including both community-based care and hospital care, patient out-of-pocket expenses, which includes the individual's own time input in the treatment process and associated travel expenses.

In terms of effectiveness, our outcome measure of choice is the Quality Adjusted Life Years (QALYs). The QALY is a composite measure of health gain which simultaneously incorporates the impact of treatment in terms of health related quality of life and life expectancy. Health related quality of life data, as measured by the Euroqol (EQ5D) questionnaire, is collected alongside the clinical trial and baseline and follow up values will be combined to estimate QALY gains for each individual, and for each treatment arm.

## Ethical Considerations

Ethical approval has been granted by the Research Ethics Committee of the National University of Ireland, Galway and the Irish College of General Practitioners (ICGP).

## Discussion

The results of this trial will be directly applicable to primary care settings in Ireland. Should a structured education pulmonary rehabilitation programme delivered at the level of the GP practice be found to be effective in improving patients' sense of dyspnoea and HRQoL then the findings would be applicable to many thousands of individuals in Ireland and beyond.

## Competing interests

The authors declare that they have no competing interests.

## Authors' contributions

KM, DC, AC, BMC, LM, DD, MN, AM and JN designed the trial protocol and secured funding for the PRINCE Trial. DD, KM and DC drafted the manuscript and AC, BMC, EoS, LM, MN, AM and JN contributed to the manuscript. All authors read and approved the final manuscript.

## Pre-publication history

The pre-publication history for this paper can be accessed here:

http://www.biomedcentral.com/1471-2466/11/4/prepub
